# Mathematical Modeling of Neuroinflammation in Neurodegenerative Diseases

**DOI:** 10.1002/psp4.70064

**Published:** 2025-08-13

**Authors:** Alex Foster‐Powell, Amin Rostami‐Hodjegan, Guy Meno‐Tetang, Donald E. Mager, Kayode Ogungbenro

**Affiliations:** ^1^ CAPKR, University of Manchester Manchester UK; ^2^ Certara, Predictive Technologies Sheffield UK; ^3^ Neuroscience R&D, AstraZeneca Cambridge UK; ^4^ Department of Pharmaceutical Sciences University at Buffalo, SUNY Buffalo New York USA; ^5^ Enhanced Pharmacodynamics, LLC Buffalo New York USA

**Keywords:** Alzheimer's disease, mathematical modeling, microglia, neurodegenerative disease, neuroinflammation, Parkinson's disease, QSP

## Abstract

Age‐related neurodegenerative diseases such as amyotrophic lateral sclerosis (ALS), Alzheimer's disease (AD) and Parkinson's disease (PD) are an increasing public health concern. Whereas the pathology of these diseases is complex, chronic central inflammation, or neuroinflammation, is commonly observed across many neurodegenerative diseases. Despite a huge wealth of resources and promising preclinical testing, effective disease‐modifying therapies do not exist. This failure is owing to a combination of poor biological understanding of this response, unsuitable animal models, and poor scaling from pathway up to clinical levels. In order to address these challenges, systems‐level mathematical models may be utilized. Here, we provide a background on neuroinflammation and summarize available mathematical models of this response. Models described by ordinary, partial, and delay differential equations, and Boolean logic are introduced and discussed. The results as discussed in this review suggest logic‐based modeling as a formalism capable of managing the challenges associated with the modeling of CNS diseases.

**Abbreviations**
AD: Alzheimer's disease
AE: adverse event
ALS: amyotrophic lateral sclerosis
APC: antigen presenting cell
APOE: apolipoprotein E
ATP: adenosine triphosphate
Aβ: amyloid beta
BACE: β‐site amyloid precursor protein cleaving enzyme
BBB: blood–brain barrier
CNS: central nervous system
CSF: cerebrospinal fluid
DAMP: damage‐associated molecular patterns
DDE: delayed differential equation
EAE: experimental autoimmune encephalomyelitis
FAD: familial Alzheimer's disease
FDA: Food and Drug Administration
HD: Huntington's disease IFN*γ* Interferon‐gamma
IL: interleukin‐
iPSC: induced pluripotent stem cell
I*κ*B: inhibitor of kappa b
KEGG: Kyoto Encyclopedia of Genes and Genomes
LOAD: late onset Alzheimer's disease
LPS: lipopolysaccharide
mAbs: monoclonal antibodies
MHC: major histocompatibility complex
MOA: method of action
MS: multiple sclerosis
NF‐kB: nuclear factor‐kappa B
NO: nitric oxide
NSAID: non‐steroidal anti‐inflammatory drugs
ODE: ordinary differential equation
OPC: oligodendrocyte progenitor cell
PAMP: pathogen‐associated molecular patterns
PD: Parkinson's disease
PDE: partial differential equation
PIN: protein interaction network
PK: pharmacokinetic
Poly(I:C): polyriboinosinic:polyribocytidil acid
QSP: quantitative systems pharmacology
ROS: reactive oxygen species
scRNAseq: single cell RNA sequencing
SOD1: superoxide dismutase 1
SS: steady state
STAT: signal transducer and activator of transcription
STRING: Search Tool for Retrieval of Interacting Genes/Proteins
TLR: toll‐like receptor
TNF‐*α*
: TUMOR necrosis factor‐alpha
TREM2: triggering receptor expressed on myeloid cells 2

## 1 Introduction

Age‐related neurodegenerative diseases such as amyotrophic lateral sclerosis (ALS), Alzheimer's disease (AD), and Parkinson's disease (PD) are becoming an increasing challenge to healthcare systems globally as lifespans increase [1]. The prognosis for these diseases is currently poor, with no effective disease‐modifying therapies available. Current treatments are only able to manage symptoms and provide, at best, a modest extension in life [2].

Despite a huge wealth of resources being directed at this challenge, therapy development lags behind many other disease areas [3]. These failures have typically been a result of poor predictive validity of current animal models and an over‐reliance on a predominant hypothesis (e.g., protein aggregation hypothesis) [4]. As understanding of the pathology behind neurodegenerative diseases increases, their multifactorial nature has become increasingly evident. The significance of metabolic dysfunction, oxidative and endoplasmic reticulum stress and dysregulation in the neuroimmune response or neuroinflammation are being appreciated [5].

**FIGURE 1:**
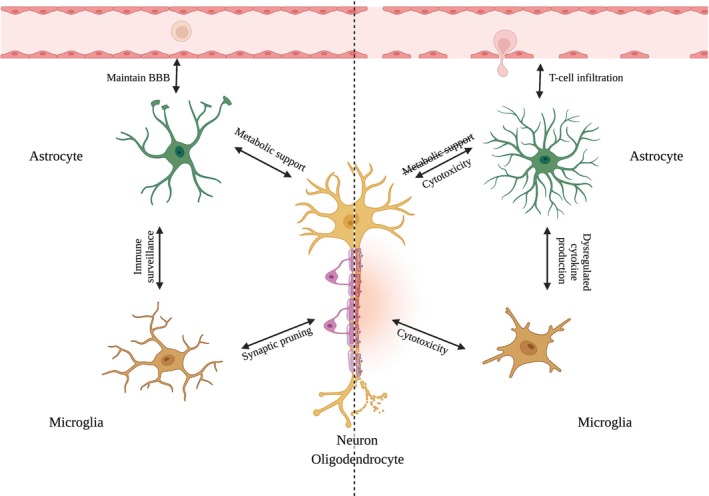
Overview of key cell types involved in the neuroinflammatory response. Created with BioRender.com.

Inflammation in the brain is an important part of health, leading to the removal of invading pathogenic species during infection or phagocytosis of debris following injury [6]. Being centrally located, this immune response must be tightly regulated, proportional, and self‐resolving once the insult has been removed. When dysregulation in the response occurs, chronic inflammation is observed, with an accumulation of pro‐inflammatory, neurotoxic species and a shift away from the homeostatic state resulting in neuronal dysfunction and ultimately death (Figure 1).

Chronic neuroinflammation has been observed as a common feature across many neurodegenerative diseases [7]. Meta‐analysis of inflammatory cytokines within cerebrospinal fluid (CSF) in patients with ALS, AD, and PD demonstrated the significant, disease specific variance in specific proteins, such as tumor growth factor‐β (TGFβ), tumor necrosis factor‐*α* (TNF*α*) and interleukin (IL)‐6, as compared to control subjects [8]. Additionally, 8‐kDa translocator protein (TSPO) positron emission tomography (PET) identified immune cell activation in the brains of living MS patients [9] and AD murine models (amyloid precursor protein (APP)/presenilin 1 (PSEN1)) [10].

With a growing body of evidence implicating neuroinflammation in many diseases, efforts have been made to characterize and therapeutically target the response. Animal models are crucial for this owing to the inaccessible nature of the CNS in humans. Animal models may be generated through genetic manipulation, as is performed in AD models (e.g., APP or PSEN1). However, these genetic mutations only represent a small minority of patients with the familial forms of these diseases; typically only 10%–20% of total cases. Alternatively, an autoimmune response may be initiated through transfer of pertussis toxin exposed cells of the spleen or lymph nodes as is performed in the experimental autoimmune encephalomyelitis (EAE) model of MS/ALS. Additionally, in vitro work, performed on isolated or cultured cells, typically from C57/BL6 mice (such as BV2 microglia), have been adopted to generate perturbation data, which provides a controlled environment to assess cellular response following stimuli in addition to cellular communication.

Many therapies targeting the immune response at the preclinical stage have appeared very promising. Minocycline, in the SOD1G37R murine model of ALS, proved able to reduce microglial activation and ultimately prolong survival by 10%–22% [11]. However, in a phase three clinical trial (NCT00047723), these results were not reproduced but in fact treatment appeared to worsen disease score in ALS patients [12]. This translatability issue implies a poor understanding of the system, insufficient knowledge of the suitability of different animal models, and inability to scale up observations at the pathway level to the clinical response.

Mathematical modeling provides a valuable resource to address some of these challenges. Traditional methods such as pharmacokinetic/pharmacodynamic (PK/PD) modeling have aimed to connect pharmacology with clinical outcomes. However, such an approach contains a few limitations; they fail to leverage existing large‐scale mechanistic details, are limited in their ability to increase understanding of the roles of components within the system, and typically only model a single or small set of biomarkers at a time (which may not represent the rate limiting step in the pathophysiology or outcome‐specific markers of the disease).

An alternative approach is quantitative systems pharmacology (QSP). This modeling approach has gained popularity since the turn of the 21st century and arose through the integration of the key concepts within systems biology and PK/PD [13]. Such QSP models are able to act as central knowledge repositories, trained to recreate experimental data, and make predictions for scenarios where no data is available. New experimental data can be incorporated into such models to provide external validation to increase the predictive confidence of such models. As such, well‐constructed QSP models are able to provide justifications for internal and regulatory decisions. Given the increased interest in neuroinflammatory responses and the rising number of therapies targeting it, it is worthwhile to assess how neuroinflammation is currently modeled and review the strengths and limitations of these models in order to identify knowledge gaps and best approaches to advance the field.

This review aims to address the current consensus and challenges in the modeling of neuroinflammatory disease and is divided into two sections. The first section introduces the biology behind the neuroinflammatory response, and section two provides a systematic review of current mathematical models of neuroinflammation in the literature, as well as the strengths and limitations of current approaches.

## 2 Neuroinflammation as a Common Feature in Neurodegenerative Diseases

### 2.1 Microglia

Microglia are the primary immune cell within the CNS and account for 5%–20% of all glial cells. Although microglia play important roles in neuroplasticity during development, they are dominantly regarded for their function in the innate immune system within the adult CNS [14, 15, 16]. Microglia are a class of macrophage that are derived from peripheral immune cells, which separate during yolk development and migrate to the developing CNS before the formation of the blood brain barrier (BBB) [17].

In homeostatic conditions, microglia survey and interact with their micro‐environment through the expansion and retraction of mobile processes and their rich assortment of surface receptors. Thus, microglia are able to detect damage‐associated molecular patterns (DAMPs) such as adenosine triphosphate (ATP) through purinergic receptors or amyloid‐β through triggering receptor expressed on myeloid cells 2 (TREM2) and pathogen‐associated molecular patterns (PAMPs) such as the gram‐negative derived surface marker lipopolysaccharide (LPS) through Toll‐like receptor 4 (TLR4) [18]. Upon receptor binding, the signal is internalized and transcription factors are activated, which ultimately governs the response, such as phagocytosis, cellular expansion and/or the production of inflammatory cytokines. These cytokines allow microglia to communicate and coordinate a response with proximal cells. Cytokine binding (e.g., IL‐6, TNF*α*, IL‐4, TGFβ) regulates the activity of transcription factors such as nuclear factor‐*κ*B (NF*κ*B) and signal transducer and activator of transcription (STAT)‐1/3, which are major regulators for the immune response as well as regulators of cell survival [19]. Positive feedback loops within these responses are known (TNF*α*, IL‐1β) with others proposed through modeling (TGF‐β) [20]. As such, this response must be highly controlled to prevent unregulated cytokine expression and neuronal death, which is often observed in neuroinflammatory disease.

Historically, microglial activation has followed similar nomenclature assigned to macrophages, divided into M1, “pro‐inflammatory” and M2, “pro‐repair” phenotypes characterized by the presence of surface markers such as CD11/16/32 and Arginase 1, respectively, and cytokine expression profiles [21, 22]. However, as transcription profiles have been generated for microglia across various neurodegenerative diseases, animal models, and experimental conditions, further distinct subpopulations have been identified such as disease associated microglia (DAM), receptor‐interacting protein kinase 1 (RIPK1)‐regulated inflammatory microglia (RRIM) [23], as well as 9 additional unnamed sub‐clusters identified in AD patient brain tissue [24]. As understanding of the different microglial phenotypes increases and with the increasing adoption of single cell RNA sequencing (scRNAseq), it appears inevitable that further biologically relevant subforms will be identified. In order to prevent this future spin off and explosion in new names, alternative naming conventions have been proposed whereby information regarding the transcriptomic or proteomic profiles under specific conditions are provided [21]. Additionally, it is currently unclear whether these represent distinct subpopulations or the degree to which one single microglia is able to transition between each state (levels of plasticity). These are one of many knowledge gaps which pose a challenge to both biologists and modelers.

### 2.2 Astrocytes

Astrocytes are the most numerous glial cells in the brain, found ubiquitously throughout the CNS, playing an accessory role promoting neuronal survival, metabolic and structural integrity, as well as maintaining the BBB through end‐feet connections [25]. Importantly, astrocytes also form part of the tripartate synapse, removing excess neurotransmitters via transporters such as GLT‐1 [26]. Astrocytes are also actively involved in the central immune response, not only expressing surface receptors allowing for their response to microglial or peripherally derived cytokines, but also their ability to propagate inflammation through the secretion of many cytokines and chemokines themselves [27]. For example, exposure of rat astrocytes in vitro to ALS patient CSF resulted in the upregulation of many pro‐inflammatory cytokines [28]. Under inflamed conditions, astrocytes appear to be both directly and indirectly neurotoxic. Transplantation of sporadic ALS patient‐derived astrocytes into the spinal cord of non‐obese diabetic severe combined immunodeficient mice resulted in motor neuron degeneration [29]. Additionally, supernatant from interferon‐gamma (IFN*γ*)‐treated human astrocytes ex vivo proved to be directly neurotoxic to SH‐SY5Y neurons [30]. Finally, astrocytes are believed to contribute to wider inflammation through their reduced ability to maintain BBB integrity [31]. Similar reduction in BBB integrity has also been observed in elderly and MS patients, potentially through a similar mechanism [32]. Astrocyte nomenclature faces many of the same challenges encountered by microglia. Traditionally, activated astrocytes were classified into A1 and A2 or neurotoxic‐vs‐neuroprotective; however, for the same reasons mentioned above, such terminology should be avoided [33].

### 2.3 Oligodendrocytes

Oligodendrocytes are primarily regarded within the CNS for their role in neuronal myelination and formation of the myelin sheath surrounding the axons of neurons, allowing for the fast saltatory conduction of action potentials [34]. The loss of oligodendrocytes and subsequent demyelination of central neurons is a hallmark of many neurodegenerative diseases, including ALS and MS. Considerable attention has been paid to microglia and astrocytes within neuroinflammation and neurodegenerative disorders; however, interest has only recently been turned to investigate the impact of neuroinflammation on oligodendrocytes. Whether oligodendrocytes are a passive bystander or actively involved in neuroinflammation is not well understood. However, it has been suggested that in ALS, neuroinflammation disrupts the maturation of oligodendrocyte precursor cells (OPCs) into terminally differentiated oligodendrocytes, potentially as a result of TNF*α* signaling [35]. Conversely, TNF*α* has also been demonstrated to encourage the generation of OPCs via TNFR2 signaling in vivo [36]. More directly, when exposed to Th1‐derived cytokines (IFN*γ*, TNF*α*, and IL‐17) oligodendrocytes upregulate many inflammatory species such as IL‐6 and CCL2 [37]. Similar transcriptional profiles have been observed in animal models for AD (5xFAD) and MS (EAE) and in AD patients [38, 39].

### 2.4 Immune Cells of the Periphery

Historically, the peripheral and central immune systems were considered as separate entities, with the central immune system being classed as “immune privileged.” However, this no longer stands true with peripheral T‐cells and monocytes/macrophages known to occupy the choroid plexus and perivascular space under physiological conditions with very few, if any, present in the parenchyma [40, 41]. With age and in pathological conditions, modest infiltration of peripheral cluster of differentiation (CD)‐4^+^ and CD‐8^+^ T‐cells is observed in both postmortem brain samples and animal models around pathological regions [42, 43, 44]. This migration is believed to be partly driven by astrocyte secreted CCL2 (also known as MCP‐1), a very potent chemokine that binds to CCR2 on macrophages and results in their migration (and drives them toward the “pro‐inflammatory” state) [45] in addition to age and disease associated mechanisms, astrocytes can drive changes in BBB permeability [46]. Although controversial, CCL2 blood concentrations have been suggested as a biomarker for cognitive decline in AD patients [47]. Members of the adaptive immune system, B‐ and T‐cells, have also been found in the postmortem brains of AD patients [44]. The specific function that monocytes and the adaptive immune system serve are not well characterized; however, it is understood that infiltrating T‐cells adopt a Th1 phenotype and drive the inflammatory response through IFN*γ* secretion [48].

### 2.5 The Pleiotropic Impact of Inflammatory Cytokines on Neuronal Survival

The direct impact of individual cytokines on neuronal health in vitro has been attempted, however, within this setting, neuronal death is difficult to accurately observe through neurons propensity to survive [49]. Therefore the impact of these messengers on neuronal survival is typically measured either with co‐cultures or in vivo. A result of this is that treatment with a single factor will inevitably lead to the expression of a wide array of cytokines, each exerting their own impact upon neuronal survival. Therefore neuronal death or survival is a complex expression of the sum of these interactions and the dynamics of the system.

TNF*α* is a complex cytokine that binds to the TNFR1 receptor, which traditionally leads to the classic downstream activation of NF*κ*B. This results in the upregulation of pro‐survival signals, such as B‐cell lymphoma 2 (Bcl‐2) and inhibitors of apoptosis (IAPs), supporting cell survival in neurons in addition to promoting pro‐inflammatory messengers in microglia and astrocytes [19]. In the disease state, however, alternative pathways may become relevant, potentially through loss of function mutations or through down regulation of species as a result of age. One such pathway is the RIPK1 pathway within TNF*α* signaling. In addition to traditional NF*κ*B activation, TNFR1 binding can also lead to the downstream activation of caspase‐8 and/or mixed lineage kinase domain like pseudokinase (MLKL), leading to cell death via apoptosis and necroptosis, respectively [50]. As a result, the direct inhibition of TNF*α* through antibodies removes these “pro‐survival” signals in neurons in addition to NF*κ*B‐mediated inflammation, thus possibly providing a justification for the failure of TNF*α* inhibitors in the clinic [51]. Downstream intermediates such as RIPK1 offer a means to separate the apoptotic/necroptotic signals from the desired NF*κ*B activation in neurons and, as such, has presented itself as a potential therapeutic target [52]. Trials targeting this pathway have been undertaken; however, agents have suffered from poor liver toxicity, and no central effects have been observed despite being CNS penetrant [53]. More recent trials in ALS patients targeting this pathway (DNL747) show low receptor occupancy and raise concerns that increasing dosage levels might lead to greater toxicity (NCT03757351). Thus, whereas understanding of the pathway in isolation has improved, the connection between target engagement and clinical outcome remains unclear.

Interleukin 6 (IL‐6) is another complex cytokine that demonstrates a pleiotropic role. Traditionally, IL‐6 is known for its pro‐inflammatory activity and exacerbating inflammation. CSF IL‐6 concentration has been suggested as being inversely proportional to disease progression in PD patients [54]. However, IL‐6 is also significant for the promotion of neuronal and oligodendrocyte survival and differentiation [55]. Interestingly, IL‐6 KO in SOD1 transgenic mice showed little change in motor neuron survival [56].

Traditional anti‐inflammatory cytokines TGFβ, IL‐4, IL‐10, and brain‐derived neurotrophic factor (BDNF) are able to exert their “pro‐survival” effects through their regulation of NF*κ*B activity within neurons. The neurotrophin BDNF, secreted from astrocytes and microglia, is able to bind to the Tr*κ*B receptor leading to downstream Ras/Mek/Erk and subsequent NF*κ*B activation [57, 58]. Alternatively, IL‐10 is able to promote neuronal survival through inhibition of Cas8 and subsequent apoptosis [59]. TGFβ activity can result in NF*κ*B activation through TGFβ‐activated kinase 1 [60]. Thus, cytokines exhibit complex pleiotropic mechanisms, which must be accounted for in order to better understand system complexity.

### 2.6 Dysregulation of Cell Crosstalk in Neuroinflammation

As previously discussed, neuroinflammation involves multiple interacting cell types, with extensive crosstalk shaping the immune response. Accumulating evidence suggests that neuroinflammation is not merely a secondary consequence of pathology but may actively contribute to the neurotoxicity of aggregated protein species. In PD, in vitro studies have shown that microglial presence is required for α‐synuclein–mediated neurotoxicity, with microglial activation preceding dopaminergic neuronal death [61]. Similarly, in AD individuals with substantial Aβ deposition may remain cognitively intact, suggesting the presence of protective genetic or environmental modifiers [62]. As these risk factors become more clearly defined, it will be essential to elucidate how they modulate inflammatory responses and disease outcomes.

Beyond canonical cytokine signaling, several homeostatic mechanisms under healthy conditions serve to regulate immune activity, protect neurons, and support the resolution of inflammation. For example, neurons and oligodendrocytes express membrane‐bound glycoproteins such as CD200L and CX3CL1 [63, 64], which bind to their respective receptors, CD200R and CX3CR1, expressed exclusively on immune cells. These ligand–receptor interactions help attenuate immune activation, thus maintaining a neuroprotective environment.

However, during disease progression (AD, PD, ALS, etc.) and aging, neuronal loss results in decreased expression of these regulatory ligands, weakening the inhibitory signaling required to constrain immune activity. This breakdown in regulation can initiate or predispose a self‐perpetuating cycle of chronic inflammation, wherein reduced immunosuppression promotes further immune activation and neuronal damage [65].

## 3 Mathematical Modeling of Neuroinflammation

Whereas understanding of the individual components within neuroinflammatory responses has increased, the ability to translate this knowledge into a disease‐modifying treatment has lagged. Increasing knowledge of cell behaviors to various cytokines and their ability to promote or inhibit neuronal survival has gradually revealed explanations for these failures. A better characterization of how the steady states of a system alter in disease and following interventions (and the subsequent use of this knowledge via modeling) would increase the chances of overcoming many of the current challenges. Simulation‐based approaches may help to achieve this goal.

### 3.1 Literature Review

In order to provide a comprehensive and unbiased view of the modeling of neuroinflammation in the literature, articles containing one of microglia, astrocyte, neuron, oligodendrocytes or the periphery in the context of neurodegenerative disease with mathematical models were systematically retrieved and studied from the PubMed database. Searching the term: (((computer simulation[MeSH Terms])) OR (Models, Theoretical[MeSH Terms]) AND “model‐based” [title/abstract] OR “model based”[title/abstract] OR “Systems biology”[title/abstract] OR “Systems Pharmacology” [title/abstract] OR “Mathematical model”[title/abstract] OR “Mathematical modeling” [title/abstract] OR “System Mathematical”[title/abstract]) AND (“Neuroinflammation” [title/abstract] OR “microglia”[title/abstract] OR “astrocyte”[title/abstract]), identified 122 publications. Of these 122 articles, ultimately 63 papers were initially selected, which included original models incorporating any feature of neuroinflammation. Of these 63 papers, 26 papers were predominantly focused on modeling the immune response and thus were included in this review (Table 1). The remaining papers were focused on modeling metabolic dysfunctions (29 papers) or disrupted proteostasis (1 paper), with 7 papers focused on other areas such as durotaxis for implants or retinal vasculature development.

**TABLE 1:** Current systems level models of neuroinflammation in the literature.

Disease	Model	Formalism	Calibration/qualification	Application/prediction	Year	Reference (PMID/DOI)
AD	Network dynamics‐based subtyping of Alzheimer's disease	Logic	Patient‐specific genomic data, single‐cell RNA sequencing data, and risk allele counts	Subtyping AD patients based on genetic and molecular regulatory mechanisms; identifying therapeutic targets	2024	39415193
AD	Aβ‐mediated synaptic glutamate dynamics and calcium dynamics in astrocytes associated with Alzheimer's disease	ODE	Glutamate uptake following in vitro exposure to amyloid	Establish a relationship between mean glutamate concentrations and Ca2+ oscillation amplitude	2024	39712135
AD	Numerical simulations and bifurcation of Ca2 + oscillatory behavior in the connection of neurons and astrocytes	ODE	Bifurcation analysis and time simulations to validate the behavior against experimental data on astrocyte signaling.	Highlights astrocytes' ability to regulate neuronal excitability, influence firing patterns, and facilitate neuronal information processing	2024	39546152
AD	TNF*α* and sphingolipid signaling pathway network model	ODE	Gene expression values from the microarray of AD postmortem brains	Predict the impact that Etanercept, Nivocasan, and Scyphostatin have on neuronal survival.	2022	36,060,699
AD	Unraveling Aβ‐mediated multi‐pathway calcium dynamics in astrocytes	ODE	Parameters selected which qualitatively captured experimental Calcium oscillations in astrocytes	Targeting of multiple calcium pathways (ion channel blockers or receptor agonists) simultaneously may restore calcium homeostasis	2021	34970118
AD	Monocyte and macrophage dynamics	ODE	Clinical data of patient peripheral monocyte and macrophage phenotype	Quantification of peripheral innate immune response changes with disease progression	2021	34,154,615
AD/PD	TNF*α* pathways in neuroinflammation, oxidative stress and insulin resistance	ODE	CSF, plasma and brain samples of human diseased and age match con‐ trolled for key species	Autocrine feedback loops continuously activate microglia and astrocytes which was enhanced by low insulin levels	2020	10.1007/s41109‐020‐00307‐w
AD	Neuroinflammation model from Puri et al. with incorporated calcium ion homeostasis	ODE	None	Effect of calcium ions on the production of amyloid‐β peptides (Aβ), microglia, and astroglia during the pathogenesis of Alzheimer's disease	2018	29754213
AD	Neuroinflammation model from Puri et al.	ODE	None	The contribution of microglia and astrocytes during the pathogenesis of Alzheimer's disease	2017	28707174
AD	PDE modeling of cell communication in AD	PDE	5% neuronal death rate at SS	Simulate the impact that mono‐ and combination therapies has on system dynamics	2016	27863488
AD	Temporal‐logic analysis of microglial phenotypic conversion with exposure to Aβ	Logic	Qualitative agreement of some model outputs with in vivo murine data	Identify signaling events which explain the difference in response observed by young and aged microglia following exposure to Aβ	2015	25406664
AD	Mathematical model for the cell–cell communication in AD progression	ODE	None	Contribution of microglia and astrocytes during the pathogenesis of AD	2010	21179474
ALS	Cell–cell communication network model	ODE	Data from the literature in a mouse model of ALS	Optimize therapeutic strategies to improve survival and quality of life	2013	23287963
Brain injury	ODE‐based model of cytokine dynamics in TBI	ODE	CSF data from patients post injury	Link between acute neuroinflammatory components and patient outcome	2018	30,563,537
Brain injury	Quantitative characterization of NF*κ*B response in microglia	ODE	Whole cell extracts quantified by ELISA in isolated BV2 mouse microglia	Model the dynamics of NF*κ*B signaling in response to TNF*α*	2011	21729324
Epilepsy	ODE‐based modeling of the neuroimmune interactions in the development of epilepsy	ODE	TNF*α* concentration, neuronal loss, seizure occurrence and BBB measurement from TMEV animal model for epilepsy	Predictions of therapeutic strategies, revealing injury‐specific therapeutic targets and optimal time windows for intervention	2022	35601918
Glaucoma	PDE modeling of glaucoma pathogenesis	PDE	None	Provide insight into the sensitivity coefficients of glia‐mediated pathology of glaucoma	2019	31496554
Gulf war illness	Logic model of neuronal‐glial interaction during neuroinflammation	Logic	Gene expression profile of a mouse model of Gulf War Illness	Understand which steady states may be settled into, and trialing the effect of pharmacological interventions on neuronal survival	2018	30374291
MS	Reaction–diffusion‐ chemotaxis model for the dynamics of multiple sclerosis	PDE	Agreement with clinical MRI data	Reproduce the molecular processes involved in the genesis of type III lesions	2017	28039494
MS	ODE‐based model for T‐cell dynamics	ODE	T‐cells from central and peripheral tissue of an EAE mouse model	Modeling T‐cell dynamics during EAE and the significance this has on disease progression	2013	23618467
PD	Dynamics of the innate immune response in PD	ODE	None	Assess the impact of two interventions on the dynamics of PD	2022	35901788
Stroke	Hsp72 regulation of NF*κ*B activation in microglial cells subjected to tumor necrosis factor‐α	ODE	BV2 mouse microglial cell line in vitro	Mechanism through which Hsp72 regulates NFkB activation, and IKK as a therapeutic target	2014	24516376
Stroke	Dynamics of microglial activation and neuronal death	ODE	Qualitative agreement with in vivo data	Identify key parameters to increase neuronal survival	2009	19884176
Non‐ specific	Microglial autocrine/paracrine cytokine interactions characterized with ODEs	ODE	Temporal profiles of cytokine release from isolated murine microglia following LPS treatment	Impact of knocking out cytokines has on TNF*α* response	2015	26440115
Non‐specific	Mathematical modeling of PI3K/Akt pathway in microglia	ODE	Optimized to fit in vitro observed relationships between Ca2+ and pAkt dynamics and ADP stimulus magnitude and duration	P2X receptor upregulation following prolonged application of agonist leads to a continual increase in Ca2 + baseline	2024	38457763

Abbreviations: AD, Alzheimer's diseases; ALS, amyotrophic lateral sclerosis; CSF, cerebrospinal fluid; EAE, experimental autoimmune encephalomyelitis; IKK, inhibitor of nuclear factor‐κB; MS, multiple sclerosis; NFκB, nuclear factor‐kappa b; ODE, ordinary differential equation; PD, Parkinson's Disease; PDE, partial differential equation; TBI, traumatic brain injury; TNFα, tumor necrosis factor‐alpha.

### 3.2 Diseases Modeled

**FIGURE 2:**
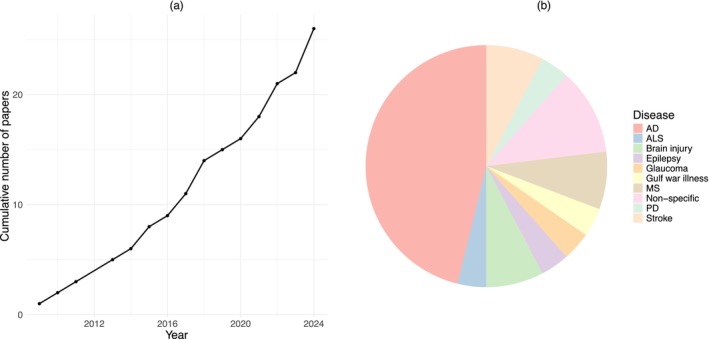
Overview of the increase in interest and applications of mathematical models in neurodegenerative diseases.

Mathematical models were found for the major neurodegenerative diseases. Of the 26 papers identified, AD was the most highly modeled (46%) followed by MS/ALS (11%), with more acute diseases such as brain injury and stroke, and PD representing 8% and 4%, respectively (Figure 2). Models of non‐neurodegenerative diseases, but still utilizing aspects of neuroinflammation, such as glaucoma and epilepsy, each accounted for 4% of total papers found.

The frequency with which diseases were modeled was roughly in accordance with relative disease prevalence, with AD most frequently and ALS the least. PD did not follow this trend; however, being considered the second most prevalent neurodegenerative disease but represented by only one paper, with MS containing the second most frequency in the literature. This, however, may be a consequence of the dominance of dopamine receptor antagonists and *α*‐synuclein in R&D pipelines in PD compared to MS, which is better known for being an immune‐mediated disease [66].

### 3.3 Common Features in Models for Alzheimer's Disease

Papers identified for AD commonly modeled the response of microglia exposed to Aβ. In an early model, an agent‐based approach was adopted, modeling plaque formation through microglial migration and activation in a 2D environment to a central supply of Aβ [67]. Later models grew in complexity to describe the pathogenesis of AD and connect microglial activation through exposure to Aβ to neuronal death, which was first demonstrated by the Puri‐Li model [68]. As the multifactorial nature of these diseases became more widely appreciated, different aspects of the disease have been integrated into these immune models, with calcium ion homeostasis integrated into the Puri‐Li model and later the interplay between oxidative stress, insulin resistance, and neuroinflammation in both AD and PD models [69, 70]. Subsequent papers focused on modeling the dynamics of cellular populations with disease course and the impact that therapeutically targeting features within neuroinflammation had on neuronal death. Treatments such as etanercept, TGFβ and aducanumab (an anti‐Aβ antibody) were commonly assessed [71]. In this case, the model identified that single therapies were inadequate in their ability to prevent neuronal death. However, a combination of etanercept and aducanumab provided modestly improved results [71].

### 3.4 Common Features in Models for Multiple Sclerosis and Amyotrophic Lateral Sclerosis

Multiple sclerosis is considered a T‐cell‐mediated autoimmune disease, and naturally models tended to contain more detail of T‐cell activation and differentiation than in other disease areas [72, 73, 74]. The work of the Villoslada group paid particular attention to connecting relapses in MS to levels of T‐cell subpopulations [73, 74]. The impact of microglia or astrocytes was not incorporated. However, greater cell communication was explored by modeling T‐cell (Treg, Th1, Th2), microglia (M1, M2) and neuronal (dead/alive) population dynamics [75]. Through sensitivity analysis, Treg and IFN*γ* concentrations were identified as key species responsible for neuronal survival. Subsequently, Treg, IL‐4, and IL‐6 dosing regimens were assessed in their ability to limit neuronal death, with the model ultimately suggesting that limiting Th1 toxicity through IFN*γ* inhibition might delay disease progression [75]. Oligodendrocytes rarely appeared in the literature surveyed. The only example of their presence was found in modeling demyelination patterns in MS [76]. This model incorporated macrophage and cytokine‐mediated induction of oligodendrocytic apoptosis. The lack of oligodendrocytes in many models represents a clear knowledge gap and opportunity, especially with their significant role in MS and ALS. This absence is largely an impact of the poor understanding of the role of oligodendrocytes within the neuroinflammatory response, which is compounded by their difficulty to culture in vitro and thus generate necessary data.

### 3.5 Common Features Across All Models

There are many conserved species that are present across all selected models. These reflect species whose behavior are well characterized and documented over many different diseases and biological models (e.g., TNF*α*, IFN*γ*). Capitalizing on this observation, Sasidharakurup et al. co‐modeled AD and PD [70]. Dependent upon the disease, protein/cell concentrations from patient CSF and serum were utilized as the initial conditions for the model. Simulations were performed to understand the interplay among inflammation, oxidative stress and insulin resistance and the change in dynamics of modeled species between the two diseases [70]. The model identified a TNF*α* feedback loop as a driver of microglial activation and glutamate production in both AD and PD. Similarly, Anderson et al. [20] identified feedback loops imposed by IL‐10 and TGFβ as crucial for maintaining model robustness. The model also suggested the presence of a TGFβ positive feedback loop, which has not been experimentally verified. Thus given the importance that these loops have in driving disease, it appears increasingly necessary to model them in sufficient detail. Incorporating and characterizing these feedback loops therefore is critical in modeling the neuroinflammatory response and may represent one of several factors contributing to the limited efficacy of monotherapies observed in the clinic.

### 3.6 Data and Model Calibration

The paucity of direct sampling of brain tissue necessitates the use of proteomic and/or RNAseq analysis of neural tissue surrounding pathological regions postmortem, offering a single time point in which to take measurements in humans. Alternatively, as an approximation to what might be occurring at the pathological region, CSF sampling or the reliance on animal/in vitro experimental models are used as a data source. Although longitudinal studies are available for ALS and AD, they typically do not evaluate neuroinflammation in any substantial detail, but rather Aβ or its metabolites [77]. The instances where longitudinal CSF samples were available were associated with acute diseases such as traumatic brain injuries over a short time‐scale (e.g., 5 days) [78]. A lack of central longitudinal data is also observed in animal/cell experimental models, making comparisons in RNAseq data between different conditions challenging. A result of this lack of longitudinal data is that currently only a qualitative description of events is available.

In contrast to in vivo systems, in vitro studies enable the modeling of intracellular dynamics. However, in systems representations of cell: cell communication, such data are rare. From the literature reviewed, only the model by Shao et al. [75] utilized longitudinal data in the modeling of T‐cell/microglia communication in ALS. Here, temporal QRT‐PCR data from mSOD1G93A/RAG2 knockout mice [79] were utilized, which generated profiles in 12 out of 20 species modeled. Parameters were subsequently fit to this dataset using a genetic algorithm method to minimize the objective function. However, a result of the paucity of data is that parameter sets may become identifiable.

The remainder of papers identified, however, were not calibrated against such a rich dataset. Some 20% of models identified in the literature review were not calibrated to experimental data (Table 1). In the PDE‐based model developed by Hao and Friedman [71], 24% (18/75) of the parameters were set based up on literature‐obtained values. The remaining majority were calibrated or fixed without fitting to experimental data. A result of this is that many assumptions must be built into the system to assign parameter values, which can limit their generalizability and validation. While not ideal, these are workarounds modelers must take in the absence of rich datasets.

Logic‐based models can provide an advantage over classical differential equation (DE) based models for large complex systems. Such models rely on far less data than standard DE‐based models and are able to be constructed with, at a minimum, a single time point [72]. Regardless of the framework employed, the outputs of these models converge in indicating that inhibiting M1 microglial activation or pro‐inflammatory cytokines is beneficial for neuronal survival [71, 72].

### 3.7 Model Framework

The majority of papers identified consisted of a series of DEs [23]. These are typically ordinary DEs (ODEs) [20]; however, depending upon the data source, partial DE (PDE) [3] and delay DE (DDE) [1] have also been utilized to include spatiotemporal dynamics of cytokine chemotaxis. Only three papers were found to use logic modeling (Table 1).

#### 3.7.1 ODE

ODE models define the dynamics of entities or state variables with respect to time and represent a common formalism across QSP and general mathematical modeling. ODEs were employed both at the subcellular (receptor binding to transcription factor activation) and cellular levels (microglia to neuron). Banaras et al. [80] developed a model for the TNF*α* mediated sphingolipid signaling pathway and simulated the impact that treatment with etanercept (anti‐TNF*α* antibody), nivocasan (caspase inhibitor), and scyphostatin (SMase inhibitor) had upon NF*κ*B activation. Interestingly neither etanercept nor scyphostatin were found to have an impact upon the model equilibrium state, a result supported by failed clinical trials for these compounds [51]. Secondly, data tended to be more available over a short time‐course at this resolution, and interactions between species are mostly well known. Such improved data availability facilitates the generation of ODE‐based models, but challenges with parameter uncertainty and model validation remain [81]. The model output may or may not become further removed from patient outcome measurements, but such modeling is excellent at characterizing transcription factor dynamics and might ultimately be used in hybrid modeling strategies to connect to clinical outcomes. However, the significance of transduction signaling must remain in context and connections to clinical effects can be very limited. Furthermore, as multiple pathways are incorporated, the amount of data that is required increases substantially, and thus there are risks of the model becoming more theoretical than clinically meaningful.

At the cellular resolution, cell activity has been modeled in a dichotomous manner; cells may be active/alive or resting/dead. The mechanism through which cytokines exert their effects may or may not be incorporated, which would be needed to assess the ability of cytokines to impact cellular phenotype. An early classic example is the Puri‐Lee model for AD [68]. Here, resting microglia were able to activate into M1 (through IL‐1 or IL‐12 and inhibited by IL‐10 and IL‐4) or M2 (through IL‐4 and IL‐10) with cells producing either pro‐inflammatory (IL‐1 or IL‐12) or anti‐inflammatory (IL‐4 or IL‐10) cytokines. Astrocytes, microglia and T‐cells were categorized in a similar manner [71]. Neurons, being the ultimate output of many of models followed suit (being dead or alive). With microglial phenotype simplified, many studies also reduced model complexity and combined cytokines together into catch‐all pro‐ or anti‐inflammatory cytokines, for cytokines that upregulate similar markers, such as TNF*α* and IFN*γ*. Subsequently, vast amounts of mechanistic detail can be lost and the specificity reduced. M1 phenotype was consistently modeled as being either directly or indirectly neurotoxic through pro‐inflammatory cytokines, increasing neuronal stress and ultimately resulting in neuronal death [68, 82]. A consequence of these reductions was that typically model output may be predicted before simulations were performed. The output of many of these models was that inhibiting these pro‐inflammatory cytokines or promoting M2/IL‐4 production would prevent neuronal death [68, 78, 82, 83, 84]. This approach ultimately failed to anticipate clinical observations in which trials for anti‐TNF*α* antibodies such as etanercept failed to alter disease progression in AD [51]. This is a helpful feature of such models, which signal the need for further research into key drug and system‐specific variables that are influencing clinical outcomes.

In order to improve upon systems modeling of different cell phenotypes, additional components must be incorporated along with their complex interplay with other species within neuroinflammatory pathways. This, however, relies on a deeper understanding of the interplay among system variables, and reliable biomarkers and experimental systems are not currently available. As an alternative, it may be acknowledged that there is a continuous number of different subforms that cells may adopt, governed by receptor binding and transcription factor activation. Such modeling has been attempted for microglia and is common in cancer models [85, 86].

#### 3.7.2 Spatial Models

A small number of papers have evaluated the inclusion of an agent‐based approach [67, 76] and PDEs [62, 66, 67, 71, 76] based approach. These spatial models were typically used to account for the chemotaxis of cells or the random diffusion of cytokines. Spatial models were implemented using an agent‐based approach in the modeling of the immune response to senile plaques [67]. This allowed for the generation of 2D plots that could be qualitatively compared to MRI patient data. Similarly, use of MRI data has been incorporated for the assessment of demyelination patterns in modeling the density of macrophages, destroyed oligodendrocytes and the concentration of chemo‐attractants [76]. Although, cell activation was not shown, the movement of these cells towards sites of inflammation was demonstrated. The ability to draw on MRI data is appealing; however, MRI data only works particularly well when focus is on physical events, such as plaque formation or demyelination. Although tools are available to detect immune system activation through MRI, these rely on markers for general cellular “activation” such as ionized calcium binding adaptor molecule 1 (Iba1) in microglia. Therefore, incorporation of such data would require the modeler to fall back into the active/inactive dichotomy that the field is trying to avoid. Additionally, inclusion of spatial data is associated with modeling limitations, such as the identification of a large number of parameters in such models, in addition to the computational burden associated with simulations [67] despite only very few state variables present, and limiting simulations to a 2D environment. Thus incorporation of MRI data to generate spatial models is currently limited by the ability to categorize cell activation and associate these with distinct, reliable and detectable markers.

#### 3.7.3 DDE

Delay Differential Equations (DDEs) function very similarly to ODEs except that they include a time delay in the interactions between species. This allows for the accounting of delays between an activating species and the activation of the effector (e.g., either the secretion, synthesis, or function of another cytokine). DDEs have been trialed in one study modeling cytokine dynamics produced by microglia [20]. The delay term was ultimately removed (set to 0) owing to the increasing computational complexity and likelihood of sharp deflections in the variables through integration issues and a lack of substantial modeling improvements. Instead, the presence of autoregulation loops was found to be critical in replicating the delayed microglial response to LPS [20].

#### 3.7.4 Logic Modeling

DE‐based mathematical models described above continuously describe the change in concentration/activity of a species with time. However, as discussed previously, this relies on a high volume of perturbation or fully quantified kinetic data to accurately parameterize. An alternative formalism is logic modeling, which instead uses discrete values for node activity governed by a series of Boolean statements. In its simplest form, node activity is simplified to the binary ON/1 or OFF/0. This simplification is biologically rooted in the typical sigmoidal “switch‐like” profiles observed in the Hill equation for ligand: receptor binding [87]. A node value of 0 does not represent the absolute lack of any of that species, but corresponds to an activity that is below a biologically relevant threshold value. Node activity is regulated by a series of Boolean logic statements, containing the logic functions AND (&), OR (—), and NOT (!).

Logic modeling may provide, at first, a parameter free space to qualitatively describe a system from reasonable defined interactions. Logic‐based models have been assessed in their ability to qualitatively recapitulate results from ODE‐based models [88]. Logic modeling has been implemented over a wide range of diseases such as irritable bowel disease to assess drug targets [89] and more recently in pancreatic cancer to describe patient heterogeneity [90]. Although this modeling formalism is being adopted in other disease areas, there are comparatively few that have been adopted for neurodegenerative diseases [90] and even fewer for neuroinflammation. The literature review performed identified three papers [72, 85, 91].

Craddock et al. [72] employed discrete 3‐valued logic states whereby node activity or expression may be below (−1), equal to (0) or above a homeostatic level (1). Nodes were updated asynchronously until the system reached steady state. Updating in this manner overcomes some of the limitations associated with temporal uniformity that is associated with synchronous model updating and is a better representation of biological variation [87]. Within the model, positive (microglia activate pro‐inflammatory cytokines which activates microglia) and negative (Neurons activate acetylcholine which inhibits neurons) feedback loops are integrated. This model utilized RNAseq data at a single timepoint over four conditions (saline, diisopropyl fluorophosphate (DFP), cortisol and DFP + cortisol). The two steady states reached by the model, homeostatic (all states = 0) and inflamed (microglia, pro‐inflammatory cytokines =1), were validated against discretized data through the construction of a Sammon's non‐linear mapping 2‐dimensional plot, which described the statistical significance of separation between measured and predicted co‐expression patterns. The activated species identified in the inflamed steady state captured those species also elevated in the cortisol/DFP and DFP treated cells (*p* = 0.0002 and 0.02). By setting the relevant nodes to −1, simulations were performed to test immunosuppressive therapy (inhibiting microglial activation), broad acting anti‐inflammatory agents (inhibiting inflammatory cytokines) and glucocorticoid receptor blockade. Whereas broad acting inflammatory agents displayed the greatest ability to return the inflamed steady state back to homeostatic conditions (88.9%), simulations demonstrated that combination of immunosuppressive therapy and glucocorticoid receptor blockade (81.8%) were more effective than each treatment individually (66.3% vs. 33.4%).

Anastasio (2015) modeled aged vs. young microglial response to Aβ or LPS treatment through declarative logic statements written in Maude, with node activity limited to integers 0–10 [85, 92]. The output of the model under these different scenarios was evaluated through propositions of species behavior (e.g., IL1b is always high if IFN*γ* is always high). Although most of the conditions tested reached a single terminal steady state regardless of the path taken, there were two instances (both with high initial IL4) where two simple steady states were identified. This highlights a limitation of the Maude methodology, whereby simulations were performed until no re‐write functions were utilized (or when the system was in a simple steady state). Thus, complex steady states (e.g., a steady state consisting of multiple cycling states) were not detected by the model, a feature that alternative methodologies are able to incorporate (e.g., BoolNet [91, 93]). Indeed, more recent work [91] has begun to overcome these limitations. Choi et al. integrated both genetic risk factors in AD as well as single‐cell RNAseq to generate patient specific probabilistic Boolean networks. Staring with a broad sampling of initial conditions, the final steady states reached by the system was clustered using UMAP to identify nine distinct patient subtypes. Following simulations in BoolNet, optimum treatment strategies were proposed for the different patient subtypes. Interestingly, even using these fairly simple models, the heterogeneous response after AKT and INPP5D activation/inhibition between patients were described. Thus, while these network may not be fully quantitative, the insights they provide can be powerful in addition to serving as valuable knowledge repositories, providing a starting protein interaction network through which future findings may be incorporated.

While logic modeling can be insightful with the lack of model parameters, the simplifications also introduce limitations over DE‐based models. The greatest limitation of logic modeling is their qualitative output. Through the binary constraints imposed on the system, graded responses, partial inhibition and establishing dose–response relationships in standard logic models become infeasible to model, all of which are typically central to pharmacometrics modeling. However, this limitation is also typically the reason why logic models are applied when early, qualitative data is only available.

## 4 Future Directions

There is a clear need for longitudinal data (e.g., disease progression biomarkers, cell populations) for these chronic diseases and more complete and unbiased repositories of protein and gene interactions to augment biological understanding. There is also a need to shift away from M1/M2 nomenclature and better definitions of microglia phenotypes based on their pertinent receptors and (micro‐) environment. Although specifics may change between diseases, there are many conserved species that are independent of disease, providing a unique opportunity to generate a multi‐purpose platform model relevant across diseases (e.g., CD200 reduction). Furthermore, as the field of neuroinflammation continues to evolve, the identification and interest in different pathways, genetic and environmental factors will inevitably shift with time. Therefore, a modular approach is required to adapt to these changes and manage model components with limited data. Logic‐based models contain unique advantages and may be utilized to generate networks describing cell differentiation, which could be inserted into wider cell communication networks. Such models may serve as a template to consider new pathways of interest or different proteins/cells to build disease‐specific constructs, such as the inclusion of TDP‐43 to produce an ALS‐specific model. However, as models increase in complexity, it will be imperative to properly test models against their key components, guided by local and global sensitivity analyses, to reliably and quickly determine the most relevant attributes and guide further experimentation and model refinement [86].

There are currently only very few examples of logic‐based models of neuroinflammation within the literature; however, it is a methodology that is increasingly being utilized within the QSP space. Pipelines exist to assist with the construction of protein interaction networks (Omnipath) and model calibration (CellNOptR). Additionally, further utility is being extracted from logic modeling through introducing rate terms for node (de)activation. Integration of time allows for dynamics to be modeled through implementing Markov processes based upon Boolean networks (MaBoSS) to extract further results from the constructed model [94, 95, 96]. Such models are also being utilized in cancer to explain patient heterogeneity in response to treatment(s) [90] and have recently been applied to AD [91]. Similar principles may be adopted as a means to characterize the differences in response between humans and animals to assist with the selection of optimal nonclinical experimental models of disease. This provides a means to reduce the high attrition rate faced by drugs targeting the CNS and capitalizing on the wealth of data compatible with logic modeling.

## 5 Conclusions

The treatment of neurodegenerative diseases poses a critical unmet medical need. Neuroinflammation is a common feature in many of these diseases, but there is difficulty in scaling what is observed at the molecular level up to alterations in disease progression or neuronal death/survival (i.e., vertical integration). While models currently exist in the literature, few incorporate the subcellular mechanistic detail that in vitro work has provided, nor the full complement of information regarding the interplay between cell types. Whereas a full biochemical QSP model would be ideal to overcome scaling issues, limitations in biological understanding and data availability make this a difficult venture. Logic models present as a promising intermediate, able to make the most of the currently available cross‐sectional data. In a modular approach, a well‐constructed cell specific logic model would be able to replicate experimental data following treatment with various stimuli in vitro. Hybrid models may be combined to bridge the gap between signaling models and in vivo clinical data. A modular hybrid approach is adaptable and could be made disease specific to include key pathways of interest. Logic models may later transition into full biochemical QSP models, to assist in the evaluation of therapies and the selection of nonclinical experimental systems. Such an approach would improve the reusability of models, provide a tool to incorporate prior clinical data, assist with identifying suitable preclinical testing and identify novel single or combined drug targets that ultimately improve candidate selection for therapeutic interventions.

## Conflicts of Interest

The authors were affiliated with the following institutions at the time of writing: Alex Foster‐Powell (University of Manchester), Guy Meno‐Tetang (AstraZeneca Neuro‐ science, United Kingdom), Amin Rostami‐Hodjegan (University of Manchester & Certara), Donald E. Mager (University at Buffalo, SUNY & Enhanced Pharmacodynamics LLC), Kayode Ogungbenro (University of Manchester). The authors declare no other conflicts of interest.
